# Improving Cognition with Nutraceuticals Targeting TGF-β1 Signaling

**DOI:** 10.3390/antiox10071075

**Published:** 2021-07-05

**Authors:** Margherita Grasso, Giuseppe Caruso, Justyna Godos, Angela Bonaccorso, Claudia Carbone, Sabrina Castellano, Walter Currenti, Giuseppe Grosso, Teresa Musumeci, Filippo Caraci

**Affiliations:** 1 Department of Drug and Health Sciences, University of Catania, 95125 Catania, Italy; grassomargherita940@gmail.com (M.G.); forgiuseppecaruso@gmail.com (G.C.); abonaccorso@unict.it (A.B.); claudia.carbone@unict.it (C.C.); teresa.musumeci@unict.it (T.M.); carafil@hotmail.com (F.C.); 2 Oasi Research Institute—IRCCS, 94018 Troina, Italy; 3 Department of Biomedical and Biotechnological Sciences, University of Catania, 95123 Catania, Italy; justyna.godos@gmail.com (J.G.); currentiw@gmail.com (W.C.); 4 Department of Educational Sciences, University of Catania, 95124 Catania, Italy; sabrina.castellano@unict.it

**Keywords:** Alzheimer’s disease, cognition, transforming growth factor-β1, nutraceuticals, medicinal herbs, omega-3 and omega-6 fatty acids, carnosine

## Abstract

Rescue of cognitive function represents an unmet need in the treatment of neurodegenerative disorders such as Alzheimer’s disease (AD). Nutraceuticals deliver a concentrated form of a presumed bioactive(s) agent(s) that can improve cognitive function alone or in combination with current approved drugs for the treatment of cognitive disorders. Nutraceuticals include different natural compounds such as flavonoids and their subclasses (flavan-3-ols, catechins, anthocyanins, and flavonols), omega-3, and carnosine that can improve synaptic plasticity and rescue cognitive deficits through multiple molecular mechanisms. A deficit of transforming growth factor-β1 (TGF-β1) pathway is an early event in the pathophysiology of cognitive impairment in different neuropsychiatric disorders, from depression to AD. In the present review, we provide evidence that different nutraceuticals, such as *Hypericum perforatum* (hypericin and hyperforin), flavonoids such as hesperidin, omega-3, and carnosine, can target TGF-β1 signaling and increase TGF-β1 production in the central nervous system as well as cognitive function. The bioavailability of these nutraceuticals, in particular carnosine, can be significantly improved with novel formulations (nanoparticulate systems, nanoliposomes) that increase the efficacy and stability of this peptide. Overall, these studies suggest that the synergism between nutraceuticals targeting the TGF-β1 pathway and current approved drugs might represent a novel pharmacological approach for reverting cognitive deficits in AD patients.

## 1. Nutraceuticals and Mental Health: Focus on Cognitive Function

The term “nutraceutical” dates back to more than 30 years ago [1]. Among the numerous definitions, González-Sarrías et al. classified nutraceuticals as “a type of dietary supplement that delivers a concentrated form of a presumed bioactive(s) agent(s), nutrient or non-nutrient, but from food origin” [2]. Vegetables, containing a variety of bioactive compounds and micronutrients able to ameliorate the health status and/or decrease the risk of developing different diseases (e.g., omega-3 fatty acids), are also considered nutraceuticals [3].

According to World Health Organization (WHO), mental health, an integral and essential component of health, is “a state of well-being in which an individual realizes his or her own abilities, can cope with the normal stresses of life, can work productively and is able to make a contribution to his or her community” (https://www.who.int (accessed on 23 June 2020)). In healthy people, cognition refers to the mental abilities allowing them to receive, acquire, and elaborate information from the surrounding environment and involving several brain areas [4]. Cognition is based on complex processes such as attention, perception, planning, learning, memory, and language [5], coordinated by executive control, emotional, and motivational components, that modulate the behavior of each individual person. An impairment of cognitive function represents a clinically relevant dimension in different neuropsychiatric disorders such as schizophrenia, depression, and Alzheimer’s disease (AD) [6].

Among the natural compounds, flavonoids and their subclasses such as flavan-3-ols, catechins, anthocyanins, and flavonols have been associated with cognitive health, and can significantly improve cognitive function through multiple molecular mechanisms [7]. Flavonoids may promote synaptic plasticity changes, influencing memory and learning processes by acting on extracellular receptor kinase, Akt (also known as protein kinase B (PKB)) and cyclic adenosine monophosphate (cAMP) response element-binding protein (CREB) [8]. Omega-3 fatty acids, essential polyunsaturated fatty acids also known as omega-3 oils, ω−3 fatty acids or *n*−3 fatty acids, and polyphenols are abundant micronutrients that are part of the human diet, and are known to exert protective effects on the central nervous system (CNS) thanks to their ability to modulate adult neurogenesis, synaptic and neuronal plasticity, promoting neuronal homeostasis and finally improving cognitive function [9,10]. Polyphenol-rich extract as an add-on to a healthy lifestyle may represent an additional pharmacological tool for the improvement of both working memory and attention [11]. Furthermore, an increased consumption of polyphenol-rich foods has been associated with better cognitive performance in elderly subjects with a high cardiovascular risk [12]. Plant-based foods, such as fruits, vegetables [13], whole-grains [14], nuts and legumes [15] are linked to many plausible effects toward human health, including brain related disorders. These effects are mediated, at least partially, through the anti-oxidant and anti-inflammatory activity of vitamins and polyphenols [10]. The synergism between nutraceuticals and current approved drugs for the treatment of cognitive disorders can be used as a novel pharmacological approach to improve cognitive function in patients with neurodegenerative disorders. For example, the co-administration of vitamin D and memantine, an N-methyl-D-aspartate (NMDA) antagonist used for the treatment of moderate-to-severe AD, improved the cognitive performance in AD patients when compared with memantine alone [16].

Carnosine is a natural bioactive dipeptide synthesized starting from its two constituting amino acids, β-alanine and L-histidine, through the activity of carnosine synthase enzyme [17,18]. This nutraceutical dipeptide, widely distributed in the tissues and organs of vertebrates [19], has been shown to possess pro-cognitive effects under both physiological and pathological conditions. The dietary supplementation of carnosine in combination with its methylated analogue anserine for more than 12 weeks has been shown to improve cognitive function [20,21], also preserving verbal episodic memory and brain perfusion [20,22], and positively modulating network connectivity cognition-associated changes [20] in elderly subjects. With regard to the therapeutic effects in cognitive disorders, carnosine gave beneficial cognitive effects in Gulf War illness [23], mild cognitive impairment (MCI) [24], and AD [25,26] subjects. In a study carried out by Fonteh et al., a selective deficit of carnosine has been linked to cognitive decline in probable AD subjects [27].

In the present review, we will focus on selected nutraceuticals that are able to enhance cognitive function by targeting a specific pathway, the transforming growth factor-β1 (TGF-β1) pathway, which exerts a key role in the pathophysiology of cognitive disorders.

## 2. TGF-β1 in Cognitive Disorders

TGF-β1 is a well-known anti-inflammatory cytokine that can act as a neurotrophic factor exerting an essential role in the initiation and maintenance of neuronal differentiation and synaptic plasticity at CNS level. TGF-β1 is able to protect neurons against the damage induced by different stimuli such as excitotoxins, hypoxia/ischemia, and amyloid-β (Aβ) aggregates [28,29].

**Figure 1:**
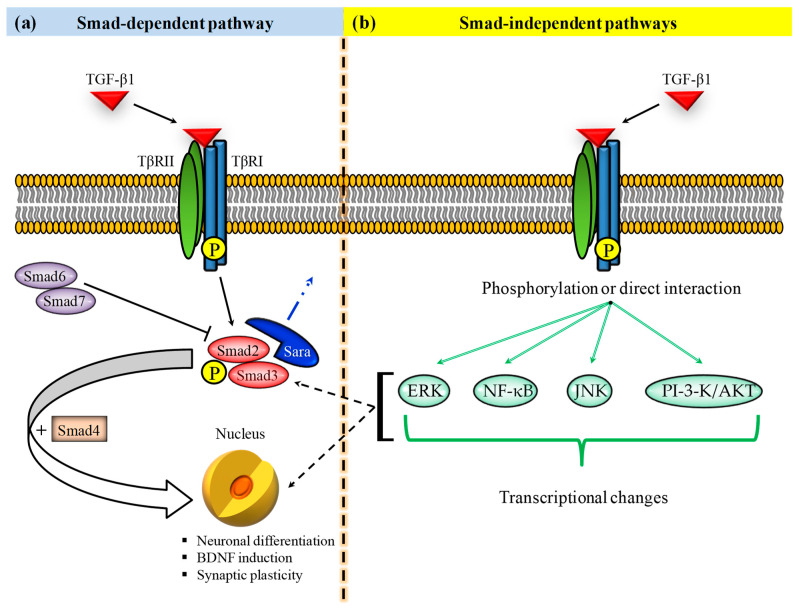
(**a**) Smad-dependent and (**b**) Smad-independent TGF-β1 signaling pathways are involved in the regulation of various cellular processes, including cell growth/proliferation, differentiation, cell migration, invasion, and extracellular matrix remodeling. (**a**) TGF-β1 binds to the TβRII homodimers allowing the dimerization with TβRI homodimers, the activation of the kinase domain of TGF-βRI kinase domain, and the phosphorylation of both SMAD2 and SMAD3. These phosphorylated proteins interact with SMAD4 leading to the formation of a heterotrimeric complex able to translocate into the nucleus with the subsequent activation or repression of different genes involved in neuronal homeostasis. (**b**) TGF-β1 can also recruit Smad-independent signaling pathways such as ERK, NF-κB, JNK, and PI-3-K/AKT. These non-Smad transducers can mediate signaling responses alone or in combination with Smads, also converging onto Smads to control Smad activities. P indicates phosphorylation. TβRII = TGF-β type II receptor; TβRI = TGF-β type I receptor; BDNF = brain-derived neurotrophic factor; Sara = Smad anchor for receptor activation; NF-κB = nuclear factor kappa-light-chain-enhancer of activated B cells; ERK = extracellular signal-regulated kinase; PI-3-K = phosphatidylinositol-3-kinase; JNK = c-Jun amino terminal kinase.

TGF-β1 signaling is initiated at the cell membrane surface through the binding of TGF-β to TGF-β type II receptor (TβRII) (homodimers) which recruits activin-like kinase 5 (ALK5)/TGF-β type I receptor (TβRI) (homodimers) forming a heterotetrameric complex with the ligand in which TβRII phosphorylates and activates TβRI [30,31] (Figure 1a).

Upon activation, TβRI phosphorylates Smad2 and Smad3 (R-Smads) that will form a complex with the Co-Smad protein Smad4 [32,33]. This complex will then translocate into the nucleus, regulating the transcription of genes involved in different cell functions such as proliferation, differentiation, and adhesion [34,35]. Not all Smad proteins are activators, in fact Smad6 and Smad7 inhibit activation of R-Smads, then inhibiting the genes’ transcription taking place at nuclear level.

In addition to Smad-dependent pathways, TGF-β1 can also activate Smad-independent pathways, including the extracellular-regulated kinase pathway [35], the nuclear factor kappa-light-chain-enhancer of activated B cells (NF-κB) pathway [36], c-Jun amino terminal kinase (JNK) pathway [37], and the phosphatidylinositol-3-kinase (PI-3-K)/ AKT pathway [38], involved in several processes such as the inhibition of cell-cycle, suppression of immune response, and neuroprotection [39] (Figure 1b). These receptor-activated, non-Smad transducers can mediate signaling responses either as stand-alone pathways or in combination with Smads, and they can also converge onto Smads to control Smad activities.

TGF-β1 plays a key role in neuronal homeostasis function axon elongation, and synaptogenesis; TGF-β1 signaling through Smad2 and/or Smad3 is also essential for maintaining quiescent microglia after injury [40]. Furthermore, this factor is able to inhibit free radical production and to induce apoptosis of stem/progenitor cells [40]. TGF-β1 can enhance synaptic plasticity by promoting the expression of brain-derived neurotrophic factor (BDNF) and tropomyosin receptor kinase B (TrkB) [41,42].

In addition to the above described, TGF-β1 has been demonstrated to be essential for the transition from early (E-LTP) to late long-term potentiation (L-LTP), underlining its role in recognition memory formation [43].

TGF-β1 knockout (KO) mice present dendritic spine density reduction and hippocampal LTP impairment [44]. The dysfunction of TGF-β1 signaling has been associated with neurodegenerative disorders; an impairment of TGF-β1 signaling has been reported in AD pathogenesis, thereby contributing to Aβ accumulation, activation of microglia as well as to the progression of neurodegeneration [42,45]. In most cases AD patients exhibit decreased levels of nuclear Smad2, Smad3, and Smad4 in the temporal cortex [46], while TβRII expression is reduced at neuronal level in an early phase of cognitive decline [45]. The double action of TGF-β1 on Smad-dependent and Smad-independent signaling is relevant when considering the pathophysiology of cognitive decline in AD and the selective impairment of Smad signaling detected in AD brains [46]. According to this scenario, the identification of nutraceuticals able to activate these Smad-independent signaling pathways and counteract the deficit of Smad signaling might be relevant for preventing AD-related cognitive decline.

Cognitive deficits are also clinically relevant in major depression, and common pathophysiological events have been identified in depression and AD, including neuroinflammation and an impairment of TGF-β1 signaling pathways [42]. Aβ injection into the dorsal hippocampus of rats has been connected to neurotoxic effects that were further amplified by intracerebroventricular (i.c.v.) injection of SB431542, a selective inhibitor of TβRI [38,47]. We have recently demonstrated a deficit of TGF-β1 signaling in a non-transgenic animal model of AD at hippocampal level [48], a brain area essential in the storage and consolidation of short-term memory that is impaired in early stages of AD [49]. Different second-generation antidepressants, in particular selective reuptake inhibitors (SSRIs), such as fluoxetine, are able to increase TGF-β1 levels in depressed patients [50] and reverse memory impairment in AD animal models [51]. It has been demonstrated that fluoxetine exerts neuroprotective effects in an in vitro experimental model of Aβ-induced neurodegeneration via a TGF-β1-mediated mechanism [52]. Furthermore, a chronic treatment with fluoxetine, or the new multimodal antidepressant vortioxetine, has been shown to completely reverse depressive-like phenotype and memory deficits in Aβ-injected mice by the rescue of hippocampal TGF-β1 levels [48]. Along this line, we can hypothesize that nutraceuticals targeting TGF-β1 signaling pathways can synergize with antidepressants to rescue cognitive function both in depression and AD.

Astrocytes represent the main source of TGF-β1 in the CNS and in the absence of pathological conditions this cell type synthesizes and releases this neurotrophic factor in different brain regions [53]. However, a TGF-β1 neuronal expression has also been reported [54,55]. In addition, TGF-β1 can be synthesized and secreted from microglia in response to inflammatory cytokines [40,56]. An in vivo study conducted by Yap et al. using an animal model of ischemic stroke [57] demonstrated that astrocytes are able to secrete Interleukin 6 (IL-6) with consequent inhibition of T helper 1 cell differentiation and the promotion of regulatory T cells (Tregs) with an increase in TGF-β1 levels contributing to the effect of hyperforin on neuroangiogenesis and functional recovery.

TGF-β1 expression and activity are primarily regulated through the conversion of latent TGF-β1 to active TGF-β1 by a variety of proteases, among which matrix metalloproteinase 2 (MMP-2) and matrix metalloproteinase 9 (MMP-9) play a central role in this conversion [58]. Psychotropic drugs, such as fluoxetine, promote the release of active TGF-β1 by favoring the activation of MMP-2 in astrocytes, and the ensuing maturation of latent TGF-β1 [52]. We can speculate that an increased conversion of latent TGF-β1 to active TGF-β1 might underlie the increased secretion of TGF-β1 by cortical astrocytes induced by some nutraceuticals such as hesperidin [59] and therefore hypothesize that cortical astrocytes might represent an ideal cellular target for natural compounds (e.g., flavonoids) [60] able to promote the secretion of TGF-β1 and then improve cognitive function.

It is known that physical activity promotes cognitive and memory functions by modulating the signaling pathway of neurotrophic factors [61] and, in turn, physical activity can exert “neuroprotective effects” after brain injury [62]. Indeed, physical activity from one hand promotes neurogenesis via synthesis and release of BDNF—one of the key neurotrophic factors involved in brain plasticity [63]—and on the other hand it increases TGF-β1 plasma levels [64], thus suggesting that physical activity can be considered as an add-on strategy to the conventional drug treatment. According to this hypothesis, a very recent study carried out by Szymura and collaborators showed that the concentrations of TGF-β1 and BDNF increased in the blood samples obtained from healthy older adults as well as in subjects suffering from Parkinson’s disease (PD) after 12 weeks of regular balance training of moderate intensity [65]. An open question remains regarding how and when a rescue of TGF-β1 levels can affect global cognitive function in these patients and what impact might be of nutraceutical targeting the TGF-β1 pathway.

## 3. Nutraceuticals Targeting TGF-β1 Pathway: Evidence from Preclinical Studies

Medicinal plants are used in traditional medical practice to alleviate or, in the best scenario, cure human suffering and illness. Medicinal plants represent a wide source of bioactive phytochemicals that play a key role in preventing chronic diseases such as cancer and diabetes [66]. In recent years, herbs have been considered an alternative approach for the treatment of neuropsychiatric disorders, and in particular of anxious and cognitive disorders, due to their good safety profile compared to current approved drugs [67].

Each class of these phytochemical compounds contains a wide range of active compounds characterized by different potencies, with some of them presenting multifunctional activity [68]. Different plants or natural compounds extracted from medicinal herbs with the potential ability to improve cognitive functions have been identified during the last decades. Among them, polyphenols, aromatic compounds isolated from fruits, vegetables, and grains have shown the ability to suppress neuroinflammation and improve memory and cognitive impairment [69]. In particular, among the subclasses of flavonoids associated with the improvement of cognitive status [7], flavonols and flavanones are able to increase the levels of TGF-β1 [70,71].

### 3.1. Medicinal Herbs

John’s wort, known botanically as *Hypericum perforatum*, is a natural agent with antidepressant activity [72], which has also recently been considered as an enhancer on cognitive function [73]. The extract of *H. perforatum* has proved to be neuroprotective in animal models of AD [74] and the hypericin, one of the most effective active compounds, is able to prevent stress-induced memory deficits and improve recognition memory induced by chronic stress in rats [75]. Hypericin promotes wound-healing phenomena [76] by inducing vascular-endothelial growth factor (VEGF) and TGF-β1 production in the burn wound area [77]. Furthermore, it has been demonstrated by Yechiam et al. that the acute administration of a low dose of *H. perforatum* (500 or 250 mg of *H. perforatum* quantified to either 1 or 0.5 mg of hypericin) has a positive effect on short-term verbal memory in healthy subjects [78]. In a different study employing an animal models of stroke, hyperforin, the main active ingredient derived from *H. perforatum*, showed the ability to promote neuroangiogenesis and functional recovery by stimulating the production of IL-4, IL-6, and TGF-β1 [57]. Both IL-4 and TGF-β1 exerted a key role in promoting the protective activity of hyperforin in post-stroke angiogenesis and recovery. In a recent preclinical study, the intranasal administration of hyperforin was able to improve post-stroke social isolation-induced exaggeration of post-stroke depression and anxiety and promoted hippocampal neurogenesis and cognitive function by rescuing TGF-β1 levels [79].

Flavonoids, a large family of polyphenolic secondary metabolites found in plants, prevent the cognitive deficits associated with chronic inflammation in vivo [80,81,82]. Flavonoid-induced activation of neuronal and glial signaling has been linked to the regulation of mammalian target of rapamycin (mTOR), vascular endothelial growth factor B (VEGF-B), and TGF-β1, promoting changes in synaptic plasticity and neurogenesis, which ultimately positively influence memory, learning and cognition [8,83]. Among flavonoids, hesperidin, a naturally flavanone glycoside present in *Citrus sinensis* [84], has been shown to improve memory performance in adult mice through increased secretion of TGF-β1 by cortical astrocytes [59]. This natural compound is also able to improve post-stroke depressive and anxiety behavior promoting neurogenesis at hippocampal level and memory function by TGF-β signaling [79]. In a different study, conducted by Li et al., a treatment for 10 days with hesperidin ameliorated behavioral impairments and neuropathology of transgenic amyloid precursor protein/presenilin 1 (APP/PS1) mice, also reducing microglial activation and TGF-β1 type 1 receptor in both the cortical cortex and hippocampus [85]. Icariin, the major constituent of flavonoids from *Epimedium brevicornum*, has demonstrated a relevant neuroprotective activity in animal models of AD as well as the ability to ameliorate the cognitive deficits induced by permanent occlusion of bilateral common carotid arteries (BCCAO) by reducing the BCCAO-induced TGF-β1 over-expression and Smad2/3 phosphorylation [86]. Icariin exerts a protective role in AD counteracting oxidative stress phenomena [87] and, most importantly, prevents memory deficits in Aβ-injected rats [88] by rescuing the BDNF signaling pathway and reverting decreases in postsynaptic density protein (PSD-95) and the phosphorylated form of cAMP response element-binding protein (p-CREB) levels. We have recently found a similar deficit of PSD-95 paralleled by a deficit of hippocampal TGF-β1 in the same animal model of AD [48]. It is also known that TGF-β1 signaling and the BDNF pathway are strictly interconnected, and that TGF-β1 enhances the expression of both BDNF and TrkB [41]. We can hypothesize that the neuroprotective effects of icariin can be mediated by an increased TGF-β1 production and the following release of BDNF, but new preclinical studies in the same animal model of AD should be conducted to validate this hypothesis.

### 3.2. Omega-3 and Omega-6 Fatty Acids

Omega-3 and omega-6 fatty acids represent two main families of fatty acids that cannot be synthesized by the human body, are therefore “essential”, and need to be introduced by the diet. Several studies have reported a positive correlation between omega-3 supplementation and a reduced risk of developing cognitive decline and dementia [89,90,91]. In addition to this evidence, it has been shown that omega-3 fatty acids are able to influence brain development and improve reference memory and mood [89,92,93]. Along this line, the reduction in omega-3 and/or omega-6 intake by the diet contributes to cognitive decline [94]. In vivo studies have demonstrated that the deficiency in omega-3 intake could also be associated with reduced biosynthesis of noradrenaline and dopamine in rat brains and then linked to a decreased learning ability [95], whereas omega-3 chronic administration improves reference memory and learning [96], and increases neuroplasticity of nerve membranes [97]. The benefit of omega-3 supplementation on cognition has also been observed in different clinical trials; in this regard, Fontani et al. have shown a positive effect of omega-3 polyunsaturated fatty acids on cognitive domains in healthy subjects, in particular, an improvement in attentional and physiological functions, particularly those involving complex cortical processing [98]. Based on the above-mentioned information, omega-3 levels and/or omega-6/omega-3 ratio could represent novel pharmacological tools for the prevention of cognitive impairment during aging and in the prodromal phase of AD [99].

Omega-3 fatty acids are characterized by an anti-inflammatory activity, as demonstrated in different studies on diseases including diabetes, arthritis, cancer, depression, and AD [100,101,102]. Lower arachidonic and docosahexaenoic acids (DHA) levels were associated with higher pro-inflammatory (e.g., IL-6) and lower anti-inflammatory (e.g., TGF-β1 and IL-10) cytokines concentrations [103]. Sharma et al. demonstrated that omega-3 fatty acids possess an inhibitory activity in ovarian cancer cells in which TGF-β1, Smad-3, and p21 levels were increased [104]. The DHA showed immunomodulatory and anti-inflammatory activities in an animal model of atopic dermatitis by increasing TGF-β1 expression and suppressing the secretion of pro-inflammatory cytokines by CD4^+^ T cells and macrophages [105]. Recently, Xu and colleagues showed a positive effect of omega-3 supplementation in a chronic renal failure animal model by regulating the nuclear factor erythroid 2–related factor 2 (Nrf2) and TGF-β/SMAD pathway [106]. The ability of omega-3 fatty acids to increase the synthesis of TGF-β1 has been shown both in vitro and in vivo [107,108]; in particular, a multicenter, randomized, double-blind, placebo-controlled trial conducted by Krauss-Etschmann and co-workers employing 311 pregnant women, long-term fish oil supplementation—containing a high concentration of omega-3 and omega-6—was associated with decreased mRNA levels of T(H)2-related molecules in the fetus and decreased maternal inflammatory cytokines, combined with an increased production of TGF-β1 acting as a master regulator in decreasing maternal inflammation [109].

Preclinical studies show that omega-3 intake is associated with an improvement in cognitive deficits paralleled by an antioxidant effect in animal models of AD, and also that the chronic administration of docosahexaenoic acid improves learning ability in aged rats [110,111]. Furthermore, a reduction in neuroinflammatory phenomena and Aβ-amyloid accumulation have been observed following the administration in vivo of omega-3 fatty acids [112,113]. Unfortunately in these studies the authors did not explore the impact of omega-3 fatty acids on TGF-β1 signaling, but recent studies in animal models of depression, a well-known risk factor for AD, support the hypothesis that omega-3 fatty acids can stimulate in vivo the secretion of TGF-β1 from microglial cells [114]. Gu et al. demonstrated that the endogenous omega-3 polyunsaturated fatty acid (PUFA) administration is able to counteract depressive-like behavior lipopolysaccharide (LPS)-induced by rescuing TGF-β1 levels and by balancing microglial M1 and M2 phenotypes [114]. Interestingly, lower concentrations of omega-3 fatty acids (in particular, eicosapentaenoic acid (EPA)) have been detected in humans in fasting plasma associated with lower TGF-β1 levels [103], and the supplementation with high dose of omega-3 fatty acids is able to reduce depressive symptoms in adolescent depressed patients [115]; despite this evidence, new clinical studies both in depressed and AD patients are needed in order to understand whether the cognitive-enhancing activity of omega-3 PUFA can be mediated by a rescue of TGF-β1.

### 3.3. Multifunctional Nutraceuticals Able to Target TGF-β1 Signaling: Focus on Carnosine and Its Therapeutic Potential in Cognitive Disorders

Among the multitude of nutraceuticals to be considered as novel therapeutic tools in improving cognition and/or counteracting cognitive disorders such as AD, recent evidence suggests a relevant therapeutic potential for the naturally occurring dipeptide carnosine (β-alanyl-L-histidine), a nutraceutical characterized by a multimodal and neuroprotective activity that includes the scavenging of reactive species [116], the negative regulation of pro-inflammatory markers [117], and the modulation of immune cells (e.g., macrophages and microglia [18,47,118,119,120]), and could thus play an important role in preventing and/or counteracting cognitive disorders often characterized by high levels of oxidative stress and neuroinflammation [121].

The ability of carnosine to modulate the activity of the above-mentioned immune cells is clinically relevant, since it has been shown that the dysfunction of both macrophages and microglia, the resident innate immune cells in the CNS, strongly contribute to cognitive decline detected in different neurodegenerative disorders such as Down syndrome, MCI, and AD [122,123,124].

With specific regard to TGF-β1, there are several studies showing the ability of carnosine to positively modulate the synthesis and the release of this pleiotropic cytokine [18,47]. In particular, in a study carried out by Fresta et al., carnosine, used at a physiological concentration (5–20 mM), was able to increase the mRNA expression levels of TGF-β1 in LPS-stimulated macrophages; this activity was also accompanied by the amelioration of the macrophage energy status, the down-regulation of the expressions of pro-inflammatory molecules and pro-oxidant enzymes, as well as the positive modulation of the expression levels of different members of the antioxidant machinery [18]. All these modulatory activities are of interest when considering different CNS disorders characterized by cognitive decline deriving from increased oxidative stress combined with neuroinflammation. Still considering the immune cells, in a model of an Aβ-induced neuroinflammation, carnosine was able to increase the gene expression levels as well as the protein secretion of TGF-β1, simultaneously preventing microglial cell death and lowering oxidative stress [47], all factors that are strictly connected to the risk of developing dementia and, more in general, to the aging-related cognitive decline [125]. In the same study, the key role played by TGF-β1 in mediating the beneficial effects of carnosine was validated by using a selective inhibitor of the type-1 TGF-β receptor (SB431542). In addition to the above-mentioned in vitro studies, the ability of carnosine to increase the production of TGF-β1, also playing a key role in hippocampal synaptic plasticity and memory [43], has also been observed in vivo in a mouse model of type 2 diabetes [126], a known risk factor for the development of AD [127,128].

When considering the therapeutic potential of carnosine, it should be also taken into account that the strong preclinical evidence is also strengthened by human studies showing an enhancement of cognition in elderly people as well as in subjects suffering of brain-related disorders [129].

All these data suggest a multimodal mechanism of action of carnosine underlying its therapeutic potential for the treatment of cognitive disorders, especially through the positive modulation of TGF-β1 production. Nevertheless, a major unmet need in this field remains that of increasing the bioavailability of carnosine both in rodents and humans after its systemic administration [125]. Carnosine administration in humans only leads to a small increase in circulating carnosine, because of its fast degradation by serum carnosine dipeptidase 1 (CNDP1) [130].

In light of the above metabolism, during the last decades lots of efforts have been made in order to develop new approaches or new formulations of carnosine able to improve its bioavailability and target delivery. A first approach might be the use of potent and selective inhibitors of CNDP1, such as carnostatine, in combination with carnosine, to increase carnosine’s bioavailability [131]. Alternatively, intranasal administration of carnosine has been proposed to bypass the blood-brain barrier (BBB) and first-pass metabolism [132,133]. Recent studies suggest that novel formulations can be developed to increase the therapeutic potential of carnosine.

#### Increasing Carnosine Delivery and Its Bioavailability: Focus on Vesicular, Nanoparticulates Systems and Derivatives

**Figure 2:**
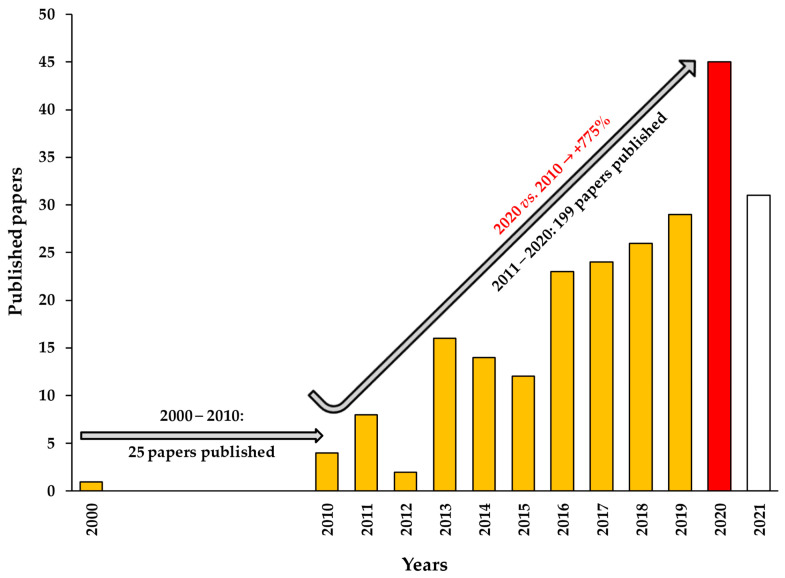
Number of published papers from January 2000 to 2021 in which the words “carnosine and nanoparticles“ were used in Science Direct (last update 1 May 2021). The production of papers from 2000 to 2010 is discontinued (one in 2000, two in 2004, four in 2005, four in 2007, three in 2008, and nine in 2009). The bar representing 2020, currently the most productive year, is highlighted in red. As clearly depicted, during the last decade (2011–2020) the number of publications has increased significantly compared to the previous ten years (2000–2010), 25 vs. 199 published papers (almost 8 times more). The current number of published papers in 2021, still in progress and indicated with a white bar, suggests that this year could represent the most productive of the last two decades.

In recent decades, the delivery of carnosine into innovative formulations (drug delivery systems) has attracted a lot of interest (Figure 2).

In particular, vesicular, nanoparticulate systems and carnosine derivatives have been investigated (Table 1).

From 2001 to 2007, the first strategy investigated was the derivatization of carnosine to increase its stability to carnosinases, representing an important limit for the therapeutic use of this molecule due to the reduction in its bioavailability [145]. Carnosine was derivatized with β-cyclodextrin and trehalose; both formulations demonstrated an antioxidant efficacy at concentrations 10–20 times lower than that reported for other synthetic derivatives [134,136]. Derivatization was also studied to facilitate the site-specific transport to different tissues. Bellia et al. investigated a new carnosine derivative (BioCar) with biotin [136]. They demonstrated an increase in the stability of this derivative towards the hydrolytic action of serum CNDP1. Moreover, the binding affinity of BioCar to avidin and streptavidin were studied with the aim to exploit the potential functionalization of gold nanoparticles.

Among the few published studies, great attention has been focused on magnetic nanoparticles coated with L-carnosine [137]. This strategy can be advantageous for its dual effect: achieving nanoparticle stability and enhancing the carnosine therapeutic effect. Peptide and/or proteins are some of the most promising materials serving as protective layers on superparamagnetic iron oxide nanoparticles (SPION) [138]. Carnosine-coated iron oxide nanoparticles have been prepared via co-precipitation of iron oxide in the presence of carnosine. The synthesized carnosine-coated magnetic nanoparticles might be applied to diagnosis and targeted drug delivery for cancer therapy [139]. Farid et al. developed stimuli-responsive magnetic nanoparticles coated with carnosine as promising nanoplatforms for breast cancer therapy. Surface grafting of magnetic nanoparticles with the dipeptide carnosine maintained nanoparticles’ colloidal stability, preventing their agglomeration. In vitro cytotoxicity results revealed superior cytotoxic effects of carnosine-coated magnetic nanoparticles on human breast cancer cell lines compared with a carnosine solution. In vivo chemotherapeutic activity on Ehrlich Ascites tumor Bagg Albino (Balb)-C mice model showed that carnosine-coated magnetic nanoparticles exhibited a significant reduction in tumor size with no observed systemic toxicity. In addition, carnosine-coated magnetic nanoparticles displayed superior anti-angiogenic effects compared with a carnosine solution [137].

Another interesting application can be found in the study of Lu et al., in which Fe_3_O_4_ nanoparticles/poly(lactic-co-glycolic acid) (PLGA) polymer-loaded dexamethasone functionalized with carnosine were prepared and investigated as a drug delivery platform for simultaneous BBB crossing and treatment of ischemic stroke. The incorporation of this dipeptide has also played an efficient role in BBB crossing transcytosis under lipoprotein receptor-related protein (LRP) receptors to access the brain tissues [140].

Among different encapsulation strategies, vesicular systems have been investigated using phospholipids, surfactants or polymers, thus obtaining liposomes, niosomes or polymerosomes, respectively [146]. In order to improve the efficacy and stability of carnosine, nanoliposomes were prepared by the thin film hydration method comparing the effects of three different lipids on the vesicles’ features (size, zeta potential, phase transition temperature and fluidity) [141]. Authors were able to demonstrate that 1,2-dioleoyl-sn-glycero-3-phosphocholine (DOPC) as well as 1,2-dipalmitoyl-sn-glycero-3-phosphocholine (DPPC) were able to provide the ideal nanoliposomes with the smallest size and highest encapsulation efficiency, probably due to the higher saturation degree compared to 1-palmitoyl-2-oleoyl-sn-glycero-3-phosphocholine (POPC).

Maestrelli et al. condensed carnosine with lipoic acid, obtaining a lipoic acid-based transient receptor potential akyrin type-1 antagonist, which was successfully encapsulated into niosomes for brain targeting [142]. Free carnosine and carnosine-loaded niosomes were investigated by in silico and in vitro studies to evaluate their effects on modifications of bovine serum albumin (BSA) and their interactions with specific amino acids [143]. Moulahoum et al. demonstrated the occurrence of a dose-dependent inhibition of advanced glycation end-products (AGE), advanced oxidation protein products (AOPP), and BSA aggregation, thus demonstrating the potential of carnosine-loaded niosomes as a valid strategy in the treatment of age-related protein modification. Recently, a novel strategy based on carnosine encapsulation in lipoprotein receptor-related protein-1 (LRP-1)-targeted functionalized polymersomes for the treatment of ischemic stroke was developed [144]. This formulation showed neuroprotective effects at a dose of carnosine three orders of magnitude lower than that of free carnosine. The LRP-1-targeted functionalization was relevant for brain targeting, allowing a time-dependent polymerosome accumulation in the brain.

## 4. Conclusions and Perspectives

Nutraceuticals deliver a concentrated form of a presumed bioactive(s) agent(s) from food/vegetables that can improve cognitive function alone or in combination with current approved drugs for the treatment of cognitive disorders. Drug discovery in the field of cognitive disorders is currently focused on the identification of active principles, with strong neuroprotective activity and high therapeutic potential. Nutraceuticals include different natural compounds such as flavonoids and their subclasses (flavan-3-ols, catechins, anthocyanins, and flavonols), omega-3, and carnosine that can improve synaptic plasticity and increase cognitive function through multiple molecular mechanisms. Rescue of cognitive function still represents an unmet need in the treatment of neurodegenerative disorders such as AD. A deficit of TGF-β1 pathway is an early event in the pathophysiology of cognitive impairment in different CNS disorders, from depression to AD.

In the present review, we provided evidence that different nutraceuticals such as *H. perforatum* (hypericin and hyperforin), flavonoids such as hesperidin, omega-3, and carnosine can target TGF-β1 signaling, increase TGF-β1 production in the CNS and finally enhance cognitive function both in rodent models of cognitive disorders and in humans. The bioavailability of these nutraceuticals, in particular carnosine, can be significantly improved with novel formulations (nanoparticulate systems, nanoliposomes, niosomes or polymerosomes) that increase the efficacy and stability of carnosine finally increasing its therapeutic potential in humans. The studies examined in the present review also suggest that the synergism between nutraceuticals targeting the TGF-β1 pathway and the drugs currently approved for the treatment of cognitive disorders might represent a novel pharmacological approach for rescuing cognitive function in patients with AD.

## Figures and Tables

**Table 1:** List of drug delivery strategies for carnosine.

	Delivery System	References
Carnosine derivatives	▪ Derivatized with β-cyclodextrins	[134]
	▪ Derivatized with threalose	[135]
	▪ Derivatized with biotin	[136]
Nanoparticulate systems	▪ Magnetic nanoparticles coated with L-carnosine	[137,138,139]
	▪ Fe_3_O_4_ nanoparticles/poly(lactic-co-glycolic acid) (PLGA) polymer loaded dexamethasone functionalized with carnosine	[140]
Vesicular systems	▪ Nanoliposomes	[141]
	▪ Niosomes	[142,143]
	▪ Polymerosomes	[144]
